# Chemokines and Innate Lymphoid Cells in Skin Inflammation

**DOI:** 10.3390/cells10113074

**Published:** 2021-11-08

**Authors:** Zhengwang Sun, Ravi Vattepu, Songfa Zhang

**Affiliations:** 1 Center for Immunology and Inflammatory Diseases, Massachusetts General Hospital, Harvard Medical School, Boston, MA 02114, USA; zsun6@mgh.harvard.edu (Z.S.); rvattepu@mgh.harvard.edu (R.V.); 2 Department of Gynecological Oncology, Women’s Hospital, Zhejiang University School of Medicine, Hangzhou 310006, China

**Keywords:** chemokines, innate lymphoid cells, skin, contact hypersensitivity, atopic dermatitis, psoriasis

## Abstract

As the outermost barrier, skin plays an important role in protecting our bodies against outside invasion. Under stable conditions or during inflammation, leukocytes migration is essential for restoring homeostasis in the skin. Immune cells trafficking is orchestrated by chemokines; leukocytes express receptors that bind to chemokines and trigger migration. The homeostasis of the immune ecosystem is an extremely complicated dynamic process that requires the cooperation of innate and adaptive immune cells. Emerging studies have been shedding a light on the unique characteristics of skin-resident innate lymphoid cells (ILCs). In this review, we discuss how chemokines orchestrate skin ILCs trafficking and contribute to tissue homeostasis and how abnormal chemokine–chemokine receptor interactions contribute to and augment skin inflammation, as seen in conditions such as contact hypersensitivity, atopic dermatitis, and psoriasis.

## 1. Introduction

The skin is a complex immune organ that functions as a barrier to protect the host from outside invasions. Anatomically, mammalian skin is composed of three layers: the epidermis, dermis, and subcutis. Skin works not only as a physical barrier but also as an immunological organ with a wide range of immune cells. These immune cells are well organized in different skin layers and establish a communication network with non-immune structural cells, such as fibroblasts, epithelial cells, and adipocytes as well as neurons, to secure a physiological barrier [1,2]. Parenchymal cells produce different kinds of cytokines and chemokines to recruit and activate immune cells, while the immune cells, in turn, secrete a range of effector cytokines, growth factors, and regulatory factors to maintain and guarantee skin homeostasis [3]. There are four groups of chemokines, C, CC, CXC, and CXC3C, which are further divided according to their amino terminal cysteine residues. At present, nearly 40 chemokines and 19 corresponding chemokines receptors have been documented [4]. Although chemokine-guided cell migration occurs to nearly all cell types, the basic and most profound function of chemokines is the recruitment of immune cells, in both inflamed and homeostatic conditions [5]. Thus, upon inflammation, immune cells expressing chemokine receptors navigate to inflamed tissue sites under the guidance of the chemokines to protect the host from pathogen [6,7]. Classically, it is well accepted that adaptive helper T (T_H_) cells are responsible for the development of several inflammatory skin disorders such as contact hypersensitivity (CHS) [8], atopic dermatitis (AD) [9], and psoriasis [10]. However, recent studies have highlighted a new subset of immune cells, referred to as innate lymphoid cells (ILCs), which directly contribute to the pathogenesis of inflammatory skin disorders [11]. In this review, we will focus on the recent understanding of how chemokine-regulated ILCs drive skin inflammation and discuss the therapeutic potential of targeting ILCs in the context of skin inflammation.

## 2. Chemokines and Chemokine Receptors

Chemokine interaction with chemokine receptors lead to different immune response-related functions, but the critical function of this interaction is chemotaxis. Generally, the entry of circulating leukocytes into inflamed tissue through extravasation includes four major steps: rolling, activation, arrest, and trans-endothelial migration [12]. Leukocyte migration occurs when chemokines are secreted by the epithelial and endothelial cells or the immune cells [13]. For example, CCL8 that had been over-expressed by skin epithelial cells and dermal myeloid cells was reported to be correlated with the expansion of skin CCR8^+^ T cells in a mouse model of atopic dermatitis [14]. Furthermore, the chemokine/chemokine receptor axis also contributes to cell retention, proliferation, differentiation, and survival. Specifically, chemokines can regulate the numbers of lymphocytes maintained in the lymph node (LN) [15]. Within the LN, T cells follow CCL19 and CCL21 gradients to interact with dendritic cells (DCs). This CCR7-dependent retention signal ends upon the expression of the sphingosine-1-phosphate (S1P) gradient, which facilitates T cell egress from the lymph node [16]. In the case of proliferation, CXCR4/CXCL12 signaling predominantly regulates the differentiation of hematopoietic stem cells (HSCs) into diverse immune cells [17,18]. Additionally, neutrophil egression from the bone marrow increases by blocking the CXCR4/CXCL12 axis, suggesting an important role of CXCR4/CXCL12 in their retention as well [19]. In terms of survival, CX3CR1 has been proven to be involved in the survival of both T_H_2 cells [20] and monocytes [21].

In allergies or other types of inflammation, multiple chemokine receptors are over-expressed in inflamed sites by resident immune cells that have been stimulated by several cytokines [13]. However, the expression of tissue-specific chemokine receptors in lymphocytes may be imprinted by DCs in the secondary lymphoid organs, where they are activated [3]. For example, lymphocytes in the mesenteric LN are imprinted with CCR9 and alpha4 beta7 (α4β7), which enable intestine-specific trafficking [22]. In the skin-draining LN, activated T cells are conditioned to specifically express CCR10, CCR4, CCR8, and CLA to facilitate skin homing [23].

## 3. Tissue Residency of ILCs

**Figure 1:**
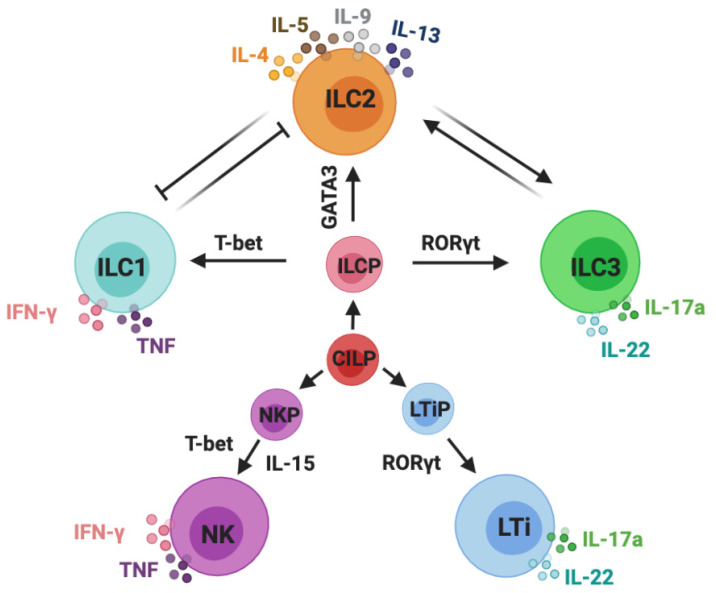
Classification of innate lymphoid cells. Innate lymphoid cells (ILCs) are derived from common innate lymphoid cells progenitors (CILP). Commonly accepted five subsets of ILCs: NK cells, ILC1s, ILC2s, ILC3s, and LTi cells. NK cells are derived from NK precursors (NKPs); ILC1s, ILC2s, and ILC3s are developed from ILC precursor cells (ILCPs); LTi cells are developed from LTi precursor cells (LTiPs). ILC1s and NK cells are categorized by their expression of T-bet and the production of IFN-γ and TNF-α; ILC2s are defined by their expression of GATA3 and are characterized by their production of IL-4, IL-5, IL-9, and IL-13; ILC3s and LTi cells are specific for their expression of RORγt and production of IL-17A and IL-22. This schematic graphic shows the classification of ILCs; however, it does not draw all of the cytokines that are required for their development and activation or indicate all of the transcription factors and immune mediators expressed by ILCs.

Innate lymphoid cells (ILCs) can be classified into five subsets: NK cells, ILC1s, ILC2s, ILC3s, and LTi cells. Group 1 ILCs comprise NK cells and ILC1s, group 2 contains ILC2s, and group 3 includes both lymphoid tissue inducer (LTi) cells and ILC3s [24]. IL-7 is essential for the development of ILC2s and ILC3s, whereas both IL-7 and IL-15 are required for the development of ILC1s. The different ILC subsets need their unique transcription factors for the development and secretion of their signature cytokines in response to different stimulators [25]. ILC1s can be activated by stimulating cytokines such as IL-12 and IL-18; for ILC2s, these are IL-25, IL-33, and thymicstromal lymphopoietin (TSLP). In the case of ILC3s, these are IL-23 and IL-1β. ILC1s are categorized by their expression of T-bet and through the production of interferon-gamma (IFN-γ). ILC2s are defined by their expression of GATA3 and are characterized by their production of IL-4, IL-5, IL-9, and IL-13. ILC3s are specific for their expression of retinoid-related orphan receptor gamma t (RORγt) and their production of IL-17A and IL-22 [26] (Figure 1).

Although the motility and tissue residency of ILCs have been discussed extensively, they remain somewhat controversial. Classic parabiosis experiments have been widely used to prove that most ILCs do not migrate from epithelial tissues in adult mice but rather proliferate in situ. For example, Sojka et al. described two populations of liver NK cells, tissue-resident NK (trNK) cells, and conventional NK (cNK) cells. Specifically, the host liver contained NK cells of host origin (CD49a^+^ DX5^−^) and NK cells derived from both the host and the donor (CD49a^−^DX5^+^), indicating that the CD49a^+^ DX5^−^ cells were trNK cells, whereas the CD49a^−^DX5^+^ cells were cNK cells. Furthermore, they showed that the features of the skin resident NK cells were similar to those of trNK cells in the liver [27]. Consistent with this, the Gasteiger team performed parabiosis and found that >95% of ILCs (including ILC1s, ILC2s, ILC3s, and LTi cells) were bona fide tissue-resident cells residing in the small intestine lamina propria, liver, and lung of the host. Surprisingly, NK cells in the lung and peripheral blood originated from both partners, suggesting a population of circulating NK cells [28,29]. Although the ILCs in the small intestine, lung, and liver have been proven to be tissue-resident cells, whether these ILCs move between tissue sites in the host remains unclear. Huang et al. first reported the existence of a subset of IL-25-responsive KLRG1^high^ inflammatory ILC2s (iILC2s) in the lung [30]. Next, they used parabiotic mouse models to address the trafficking of iILC2 recirculation. Surprisingly, they found host iILC2s (IL-25-induced) distributed in the lung, liver, and spleen of donor mice (naïve), indicating a population of circulating ILC2s. On the contrary, the IL-33-induced ST2^+^ natural ILC2s (nILC2s) did not circulate between parabiotic mice, suggesting a population of tissue-resident ILC2s [31]. Taken together, IL-25-induced iILC2s are circulating ILC2s, but IL-33-activated nILC2s are tissue-resident cells. Given that NK cells are the circulating cells of group 1 ILCs and since iILC2s are circulating subsets among ILC2s, we are curious as to whether there will be circulating ILC3 subsets; this needs further investigation in future research.

In trans-well chemotaxis assays, it was shown that neither CCL1 nor CCL8 (CCR8 ligands) exhibited chemotactic activity for ILC2s. By contrast, CCL22 (ligand for CCR4) significantly induced both WT and CCR8^−/−^ ILC2s migration but not CCR4^−/−^ILC2 migration. To confirm this in vivo, they adoptively transferred fluorescence-labeled WT, CCR4^−/−^, and CCR8^−/−^ ILC2s to recipients and analyzed their homing capacity to the lung. Their light-sheet microscopy results showed equal distributions of WT and CCR8^−/−^ ILC2s but a significantly decreased number of CCR4^−/−^ ILC2s in the lungs. In summary, these results suggest that CCR8, in comparison to CCR4, was dispensable for ILC2s migration [32]. However, other research has confirmed that the CCL8–CCR8 axis played an important functional role in ILC2 accumulation and migration in vivo under inflammatory conditions using intravital imaging [33]. Collectively, these findings indicate that the CCR4–CCL17/CCL22 axis could be more important in driving ILC2s migration, while the CCR8–CCL1/CCL8 pathway might be dispensable for ILC2 migration to the lung under certain inflammatory conditions. However, whether CCR8 contributes to ILC2 homing to skin needs further study. It has been reported that it is predominantly CCR10^+^ ILCs that migrate from the skin-draining LN into the skin under the steady state [34]. To investigate whether ILCs were self-maintained in the skin, parabiosis was performed [35]. Interestingly, the results showed that 90% of the ILCs were host original, suggesting that ILCs were mainly maintained locally in the skin at steady state. Furthermore, they treated parabionts with MC903 to introduce inflammation. The most majority (>95%) of expanded ILCs remained of host origin in the skin [35]. Taken together, the population of ILCs are maintained by self-renewal resident ILCs, with only a small contribution of circulating or bone marrow-derived progenitors under the steady state. Upon systemic perturbation, the circulating NK cells and iILC2s could migrate under the guidance of different tissue-homing markers to different peripheral sites, such as the skin, lung, and gut, where they contribute to local immunity, homeostasis, and tissue repair.

### 3.1. ILCs in Skin

Despite the broad study of ILCs in the lung and intestine, skin ILCs are far less well characterized. It was reported that Lin^−^ Thy1.2^+^ ILCs in the different anatomical layers (epidermis, dermis and subcutis) exhibited distinct phenotypes. Unlike the subcutaneous ILCs with a high uniformity of ILC2s, epidermal and dermal ILCs each contained ILC2s and ILC3s, respectively. Tbx21, a transcription factor expressed by ILC1, was undetected [35]. Upon PMA and ionomycin stimulation, the majority of subcutaneous ILCs produced the cytokines IL-5 and IL-13, which were consistent with ILC2s, while the dermal ILCs expressed IL-5 and IL-13 as well as IL-17A, indicating a heterogeneous population of ILC2s and ILC3s [35]. Interestingly and in contrast to other skin ILC subsets, epidermal ILCs only minimally produced IL-5, IL-13, and IL-17A upon PMA and ionomycin stimulation [35]. To better dissect the heterogeneity of skin ILC subsets, singe-cell RNA-seq was performed. Subcutaneous ILCs highly expressed gene profiling of ILC2, while epidermal ILCs were enriched in ILC3 signature genes, including their key transcription factor Rorc. They also expressed ILC2-associated genes, such as *Il2, Hlf*, and *Il13*. However, none of the skin ILCs expressed NK cell- or ILC1-related genes such as *Tbx21, Eomes, Ifng,* and *Il15ra* [35]. Collectively, in contrast to the typical ILC2 signature in subcutis ILCs, epidermal ILCs exhibited an ILC3 signature with skin-specific transcriptome features. Taken together, different layers of skin ILCs showed distinct transcriptome and regulome landscapes, suggesting a high degree of layer-specific identities [1,35].

### 3.2. NK Cells and ILC1s

Group 1 ILCs include ILC1s and natural killer (NK) cells. NK cells are bloodstream circulating cytotoxic cells that are dedicated to killing virus-infected cells and tumors [24]. ILC1s are generally cytotoxic and function as the first line of defense against infections with viruses and certain bacteria [36,37]. NK cells and ILC1s share the common innate lymphoid progenitor (CILP) but different precursors. Specifically, NK cells develop via the NK cells precursor (NKP), whereas ILC1s develop via the innate lymphoid cell precursor (ILCP) [38,39,40,41]. Although they develop distinctly, they share some commonalities [42,43]. For example, they both express the transcription factor T-bet and promote type 1 immunity, which are critical for controlling intracellular microbial infections and viral infections and for restraining tumor development [44,45,46]. Unlike other ILCs that require IL-7R signaling for their development, group 1 ILCs are much more dependent on IL-15 [36]. Functionally, ILC1s and NK cells are recognized as the innate counterparts of T_H_1 cells due to their high expression of IFN-γ. ILC1s and NK cells can be activated by macrophage and dendritic cell (DC)-derived interleukin (IL)-12 and IL-18 [47]. In the steady state, ILC1s and NK cells persist in the dermis at a low frequency; however, they increase dramatically when exposed to intracellular pathogens.

### 3.3. ILC2s

Unlike ILC1s, ILC2s are reliant on the transcription factors GATA3 and RORα and produce type 2 cytokines, such as IL-4, IL-5, IL-9, and IL-13, in response to tissue-derived cytokines such as IL-33, IL-25, and TSLP [48,49]. Under helminth or parasitic worm exposure in mice, ILC2s rapidly increase and secrete large amounts of T_H_2 cytokines to expel these invasive parasites [50]. Presently, the pivotal role of ILC2s in allergic inflammation in multiple barrier organs is well established in several animal models [51,52]. In addition, ILC2s maintain the homeostasis of the microenvironment in adipose tissue in mice by altering the type 2 immune environment [53,54]. Moreover, ILC2s contribute to cutaneous wound healing in mice in an IL-33-dependent manner [55].

### 3.4. ILC3s and LTi Cells

ILC3s commonly depend on the transcription factor RORγt and are mainly distributed at mucosal sites in response to extracellular bacteria and intestinal commensals [56]. ILC3s act on intestinal epithelial cells and modulate resistance to intestinal infections via the expression of IL-22, which is dependent on aryl hydrocarbon receptor (AhR) [57,58]. ILC3s also respond to IL-1β and IL-23 to produce IL-17, granulocyte-macrophage colony-stimulating factor (GM-CSF), or tumor necrosis factor-alpha (TNF-α) [59]. Accumulating evidence has shown that ILC3s play a more nuanced role in the maintenance of the symbiotic relationship with intestinal microbiota [60,61] as well as in inflammatory conditions such as psoriasis [62] and inflammatory bowel disease (IBD) [63].

Similar to ILC3s, LTi cells depend strictly on the transcription factor RORγt, but their distribution and functions are distinct [64]. LTi cells mediate the development of lymphoid tissues during embryogenesis via the production of lymphotoxin [65], while predominant ILC3s produce IL-22. LTi cells express c-Kit and CCR6 but not natural cytotoxicity receptors (NCRs) [66].

## 4. Chemokines and ILC1s in Allergic Contact Hypersensitivity

Contact hypersensitivity (CHS) is one of the most common skin diseases, affecting 15–20% of people worldwide [67]. CHS is a delayed-type hypersensitivity response that is triggered by the penetration of low-molecular-weight chemicals or metals. The chemokine–chemokine receptor system plays a critical role in the development of CHS. The CXCR3–CXCL9/CXCL10/CXCL11 axis has been reported to be the most exclusively activated signaling pathway in CHS in both human and mouse models [68,69,70]. Skin patch testing showed that CXCL10 was the most abundant and predominantly expressed by epidermal cells (mostly keratinocytes), whereas CXCL9 was expressed in both the epidermis and the dermis [68,70]. The CCR4–CCL17/CCL22 system has also been implicated in CHS. CCR4 was proved to be globally expressed on skin-homing memory T cells and was correlated with the cutaneous inflammatory T-cell response [71,72]. Moreover, CCL17, the ligand of CCR4 promoted integrin-dependent adhesion of skin memory T cells to the cell adhesion molecule ICAM-1, causing their rapid arrest [72]. A human study found that the production of CCL17 was boosted 50 times and that CCR4 increased 6-fold in nickel-induced skin inflammation compared to normal skin [73]. In addition, the production of CCL17 was increased in the epidermis of CHS lesions [74], while the serum CCL17 levels were reported to be connected with atopic dermatitis [75]. Interestingly, the roles of CXCR3 and CCR4 in mouse models of CHS were not in line with those in human samples, as increased inflammation was observed in both CXCR3 and CCR4 knockout mice [71,76]. However, the mechanism needs further investigation.

Chemokines, and chemokine-recruited immune cells, have been extensively studied and have been shown to play important roles in CHS [77]. Since ILCs have become an increasingly popular research interest, some fundamental views on the pathogenesis of allergic contact dermatitis have changed significantly. Here, we will discuss the recent reports of ILCs in the development of CHS. One study showed that CD56^high^CD16^−^CD62L^−^NK cells in allergic contact dermatitis contributed to accelerated allergic responses. In inflamed skin, NK cells gain a set of CXCR3, CCR6, and CCR5 chemokine receptors rather than CD62L and CCR7 (homing to the lymph nodes). NK cells sorted from peripheral blood mononuclear cells (PBMCs) of nickel-allergic donors failed to proliferate, be activated, and release cytokines when exposed to nickel again in vitro, suggesting that NK cells do not exhibit memory-like properties to nickel exposure in vitro [78]. However, there is a recent idea that antigen-specific or nonspecific innate immune memory may contribute to hapten-induced CHS that has emerged as a novel concept. For example, NK cells provoked hapten-specific memory responses in Rag^−/−^ mouse (lack of T and B lymphocytes) CHS models, suggesting that there were memory-like properties in the NK cells or ILC1s that were independent of T and B cells [79,80]. The Ly49 family receptors were reported to play a critical role in NK cell memory responses, not only in the sensitization phases but also in the challenge phases [81]. Moreover, IL-7Ra^+^ ILC1s acquired hapten-specific memory potential in skin-draining LNs and migrated to the liver via CXCR6, maintaining their long-term homeostasis through IL-7R signaling [82].

**Figure 2:**
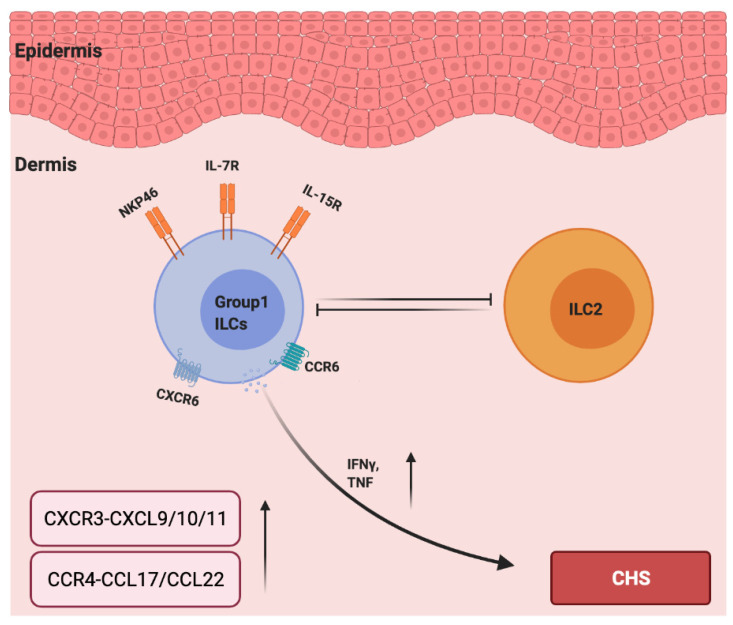
The roles of chemokines and group 1 ILCs in the pathogenesis of allergic contact hypersensitivity. IL-7 and IL15 are required for the development of group 1 ILCs. CCR6 and CXCR6 are essential for NK cell homing to inflamed skin, and NKP46 is a major activation receptor of NK cells. NK cells negatively regulate ILC2s, and ILC2s counter regulate type 1 immune responses (IFN-γ and TNF-α) and are mainly driven by group 1 ILCs. The levels of CXCR3–CXCL9/10/11 and CCR4–CCL17/CCL22 are upregulated in the lesions due to CHS.

Interestingly, unlike type 1 ILCs, ILC2s acted as negative regulators in a mouse CHS model as the ILC2-deficient mice (Rorα^sg/flox^ Il7r^Cre/+^) displayed increased ear swelling responses to TNCB challenge [83]. Moreover, a depletion of NK cells led to increased ILC2 numbers and cytokine expression in the early stage of papain-induced lung inflammation in mice, indicating that NK cells counter-regulated the ILC2s [84]. In addition, in hapten based CHS models in IL15^−/−^ mice (NK cell-deficient mice), ear swelling was reduced and was accompanied by increased numbers of ILC2s in the skin and skin-draining lymph nodes [83]. Therefore, the development of CHS could be due to the imbalance between type 1 and type 2 immunity, in which NK cells negatively regulate ILC2s, and ILC2s counter regulate type 1 immune responses that are mainly driven by NK cells and T_H_1 cells. However, how ILC1s and ILC2s influence the mutual counterbalance between type 1 and type 2 immune responses in allergic CHS remains unknown (Figure 2).

## 5. Chemokines and ILC2s in Atopic Dermatitis

Atopic dermatitis (AD) is the most common skin disease in the world among children and is characterized by dryness, redness and itching. Specifically, it is present in 60% of babies younger than 1 year, and it increases to 85% among children younger than 5 years of age [85]. Although this prevalence decreases with age, the mechanism behind this phenomenon remains unclear.

An impaired epidermal barrier is a hallmark of AD. The alteration of the epidermal structure protein filaggrin is strongly associated with the development of AD. In AD skin, elevated trans-epidermal water loss (TEWL) and a higher prevalence of invasions lead to the abnormal infiltration of immune cells, including eosinophils, mast cells, and lymphocytes (generally of the T_H_2 subtype) [86]. Numerous receptors have been reported to be important in AD. For example, CCR10/CCL27 interactions were proven to alleviate skin inflammation via regulating T cells. Specifically, in vivo, the intracutaneous injection of CCL27 attracted lymphocytes, and conversely, the neutralization of CCL27–CCR10 interactions inhibited lymphocyte trafficking to the skin, leading to the suppression of allergen-induced skin inflammation [87]. CX3CR1 was reported to be important for maintaining CD4^+^ T cells in inflamed skin. In CX3CR1-deficient mice, AD immune response and inflammation were dramatically attenuated compared to normal mice [88]. In acute AD mice skin, increased numbers of eosinophils, mast cells, and CCR4-expressing Th2 cells were confirmed, as were elevated levels of IL-4, IL-17A, IL-22, CCL17, CCL22, and CCR4 [89]. As expected, mice with CCR4-deficiency or that had been treated with a CCR4 antagonist exhibited decreased allergic immune responses, indicating that CCR4 played a crucial role in AD pathogenesis via the recruitment of CCR4-expressing Th2 cells and Th17 cells [89]. Our recent study suggested that the CXCR4/CXCL12 signal is increased in both AD patients and mouse models. CXCR4^+^ memory NKT cells preferentially resided in CXCL12-rich areas and participated in the development of AD [90]. Thus, chemokine and chemokine receptors might function as signals for the recruitment of corresponding immune cells to the inflammatory skin sites, leading to the initiation and amplification of AD [91].

CCL18 is one of the top chemokines that is over-expressed in chronic inflammatory diseases, and CCR8 functions as the CCL18 receptor in humans [92]. Mice that were deficient in CCL8 (a CCR8 ligand) exhibited decreased chronic AD-like inflammation due to an inability to recruit CCR8-expressing inflammatory T helper type 2 (T_H_2) cells or CCR8-expressing inflammatory TH2 cells enriched for interleukin (IL)-5. Additionally, the adoptive transferring of TH2 cells proved that CCR8 played a key role in recruiting TH2 cells into the allergen-inflamed skin of AD mice. In humans, CCR8 expression also highlighted a population of IL-5-enriched TH2 cells [14]. Moreover, AD patients with a blood natural killer (NK) cell deficiency and a deficit of murine NK cells (IL-15^−/−^ mice) showed enhanced AD inflammation in the skin, suggesting that an immunotherapy strategy for AD treatment could be promising [93]. Indeed, the exogenous administration of the IL-15 superagonist boosted NK cells and relieved AD-like inflammation in mice [93]. Increased populations of ILC2s and eosinophils were observed in skin lesions of IL-15^−/−^ mice compared to controls. At a steady state, IL-15^−/−^ mice also showed an elevated percentage of ILC2s, suggesting that NK-ILC2 interactions may contribute to homeosis. Furthermore, NK cells reduced ILC2 frequencies during the induction of AD using both WT and Rag1^−/−^ mice [93]. In summary, NK cells limit ILC2-manipulated type 2 inflammation in mouse AD skin. However, the exact mechanism of NK cells and ILC2s counter-striking of each other remains an open question.

**Figure 3:**
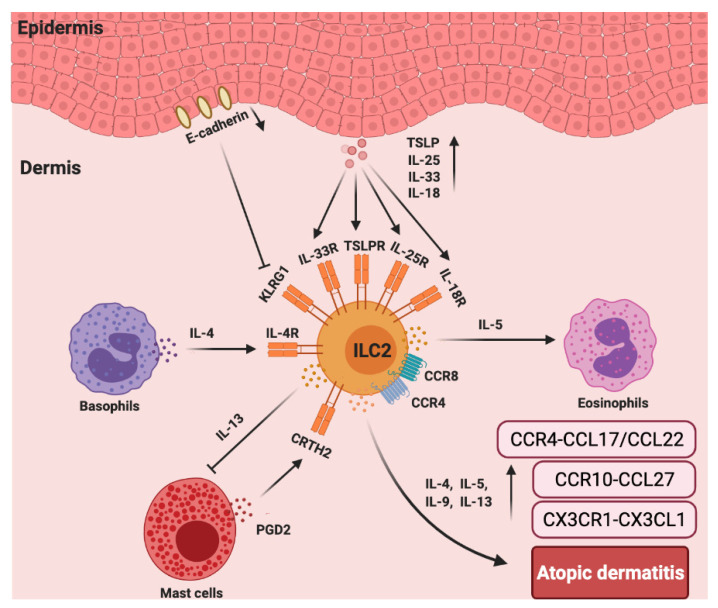
The roles chemokines and group 2 ILCs in the pathogenesis of atopic dermatitis (AD). In AD skin, there are more ILC2s and increased TSLP, IL-25R, IL-33R, IL-18R, and CRTH2 expression in lesioned skin from AD patients compared to that of healthy controls. ILC2s, basophils, and eosinophils colocalize in AD skin lesions. Basophils-secreted IL-4 induces ILC2s accumulation, and ILC2s-secreted IL-5 recruits eosinophils that accelerate AD inflammation. CCR4 and CCR8 are also important for the recruitment of ILC2s to the lesioned skin site. PGD2, which is produced by mast cells, binds to CRTH2 to induce migration and type 2 cytokine (IL-4, IL-5, IL-9, and IL-13) production by ILC2s. ILC2-expressed IL-13 can abolish IgE-dependent cytokine release by mast cells. Furthermore, E-cadherin production by keratinocytes is decreased in AD skin, which impairs the negative regulation of ILC2s via KLRG1. The levels of CX3CR1–CX3CL1, CCR10–CCL27, and CCR4–CCL17/CCL22 are significantly increased in the AD skin.

In addition to chemokines, cytokines are also important for the function of ILC2s in AD [91]. As reported, IL-2R, IL-7R, IL-18R, IL-25R, IL-33R, TSLP, IL-4, and IL-13 are linked to the activation or function of ILC2s [94]. In vitamin D3 analog calcipotriol (MC903)-induced AD-like inflammation in Rag1^−/−^ mice, the depletion of ILC2s attenuated skin inflammation [95]. Transgenic mice over-expressing IL-33 (hK14mIL33tg) exhibited an increase in IL-33R^+^ ILC2s and developed atopic-like dermatitis in the skin [96], while the genetic knock out of IL33R or IL-25R significantly reduced ILC2 response [97]. The disruption of TSLP (Tslpr^−/−^) signaling significantly limited ILC2 responses and decreased skin inflammation [95]. The cytokines involved in the activation of ILC2s in the skin appear to be much more complex than previously appreciated. Unbiased transcriptomic analysis of ILC2s sorted from the skin, bone marrow, lung, fat, and gut were performed. Particularly, skin ILC2s preferentially expressed the IL-18 receptor 1 (IL-18R1) and responded to IL-18 treatment in vitro in the presence of TSLP. In MC903-treated IL-18 deficient mice, the accumulation of ILC2s and eosinophils was reduced [98]. Consistently, mice that were over-expressing murine IL-18 (KIL-18Tg) exhibited type 2 immune responses and dermatitis-like skin [99]. However, the involvement of ILC2s was not assessed in this study. Whether IL-18-activated ILC2s contribute to this inflammation requires further investigation (Figure 3).

## 6. Chemokines and ILC3s in Psoriasis

Psoriasis is a chronic inflammatory skin disease that is characterized by well-demarcated red, scaly plaques [100]. Histologically, this T_H_17 cell-associated inflammatory diseases result in acanthosis, parakeratosis, and neutrophilic inflammatory infiltration. Classically, conventional CD4^+^ T_H_17 cells are believed to produce the effector cytokines IL-17 and IL-22 and are believed to trigger the pathogenesis of psoriasis in humans [101]. CCR4^−/−^ mice showed significantly decreased psoriasis-like inflammation compared to wild-type mice due to the reduced infiltrating T_H_17 in the psoriasis skin lesions and during the draining of the LN. When the mice were treated with compound 22, the psoriasis-like skin inflammation was significantly alleviated, with a significant decrease of the Th17 cells. CCR4 plays a role in psoriasis development via the regulation of Th17 cells [102]. Another study suggested that the CCR6–CCL20 pathway was important in recruiting inflammatory cells to the site of psoriasis [103]. The level of CCL20 was one of the top three upregulated proinflammatory cytokines in the serum of psoriatic patients, suggesting a promising biomarker for diagnosis and treatment [104]. Consistently, elevated levels of CCR6–CCL20 have been confirmed in mice with psoriasis-like skin [105]. Intradermal or subcutaneous injections of IL-23 contributed to a significant over-expression of TNF-α and IL-17A, leading to psoriasis-like inflammation. However, mice with CCR6 deficiency failed to respond [106]. Taken together, CCR4 and CCR6 are important for the pathogenesis of psoriasis.

Surprisingly, in a psoriasis-like mouse model, it was reported that T_H_17 cells were not the dominant source of IL-17 and IL-22, rather the RORγt^+^ gamma delta T (RORγt^+^ γδ T) cells and ILC3s were. Consistently, Rag^−/−^ (lack of T and B cells) mice developed psoriatic skin similar to that of WT mice with T_H_17 cells. Furthermore, Rag2^−/−^ gamma c-deficient mice (Rag2^−/−^ γc^−/−^, T, B and ILC deficient) showed decreased disease severity [107]. Collectively, these findings suggest that IL-17- and IL-22-producing ILC3s could directly contribute to the pathogenesis of psoriasis. Indeed, it was reported that the numbers and frequency of IL-22^+^ RORγt^+^ CD56^+^ ILC3s were remarkably higher in the skin from psoriasis patients relative to healthy skin [108]. In line with this, IL-17- and IL-22-expressing NKp44^+^ ILC3s were identified in the blood and skin of psoriasis patients and were preferentially harbored in lesioned psoriasis skin [109]. Taken together, these studies provide compelling evidence for the pathogenic role of ILC3s in psoriasis. However, more investigation is required to determine the relative contributions of T_H_17 cells and ILC3s in human psoriasis.

**Figure 4:**
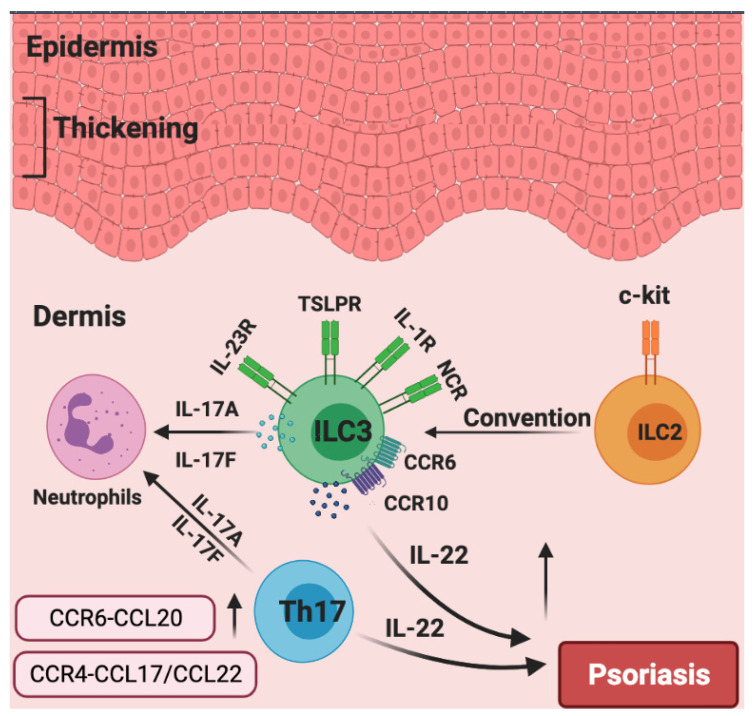
The roles of chemokines and group 3 ILCs in the pathogenesis of psoriasis. Generally, the over-production of IL-22 leads to epidermal thickening in psoriasis. Patients with psoriasis have increased numbers of NCR+ ILC3s. CCR6 and CCR10 are indispensable for the migration of ILC3 to inflamed skin. Skin-invading populations of ILC3s rather than T helper 17 (TH17) cells are the major source of IL-17A and IL-17F, which induce neutrophilic inflammation. c-Kit+ ILC2s could potentially be converted into IL-17A-expressing ILC3s. The production of CCR6–CCL20 and CCR4–CCL17/CCL22 are significantly increased in the psoriasis skin.

Given the above, focusing on ILC3s may have tremendous potential for psoriasis treatment. For example, it was reported that murine skin ILCs universally expressed CCR6 and CCR10, which guided their homing and localization under homeostatic conditions [34,106]. Moreover, CCR6 played an important role in IL-23-induced psoriatic dermatitis in mice [103], and ILCs infiltrated into the inflamed skin in a CCR6-dependent manner in MC903-induced mouse skin [110]. Of note, single-cell RNA sequencing and fate-mapping analysis on ILCs in the IL-23-induced mouse model of psoriasis showed that a group of quiescent ILCs could become activated ILC2s and could be further converted into IL-17- and IL-22-producing ILC3-like cells upon IL-23 stimulation [111]. In human skin, patients with psoriasis expressed a higher level of CCL27, a ligand of CCR10, compared to healthy controls, indicating that the CCR10/CCL27 signal facilitates the development of psoriasis [112]. It was revealed that iILC2s expressed both GATA3 and RORγt^+^ and co-expressed IL-13 and IL-17 under certain culture conditions in vitro, indicating that iILC2s could have the capacity to develop into IL-17 expressing ILC3s [30]. Consistently, c-Kit and CCR6 showed a RORγt+ ILC2 subset that can readily transdifferentiate into IL-17-producing ILC3-like cells. scRNAseq analysis identified skin-homing RORγt+ ILC2s in peripheral blood that express how CCR6 and CCR10 might contribute to IL-17-mediated pathologies [113]. Furthermore, a profound increase in ILC3s and a decrease in ILC2s were observed in the skin from psoriasis patients relative to healthy skin, the frequencies of IL-17-producing ILC3s were increased at the expense of ILC2s within the lesioned skin of patients with psoriasis [108], suggesting c-Kit+ ILC2s could turn into IL-17-producing ILC3s during the pathogenesis of psoriatic inflammation. Collectively, these studies highlight that targeting CCR6 and/or CCR10 to inhibit the infiltration of ILCs into the skin could be a tremendously promising therapeutic alternative in psoriasis. We also speculate that the dynamics and plasticity of skin ILCs in response to IL-23 might shed more light on psoriatic inflammation upon further investigation (Figure 4).

## 7. Concluding Remarks

In this review, we discussed the role of chemokines and ILCs in the development of skin inflammatory diseases. Specifically, the CXCR3–CXCL9/CXCL10/CXCL11 and CCR4-CCL17/CCL22 systems play important roles in CHS development; the CCR10–CCL27, CX3CR1–CX3CL1, and CCR4–CCL17/22 pathways are related to the pathogenesis of type 2 inflammation in AD skin, and the CCR4–CCL17/CCL22 and CCR6/CCL20 axis show promising potency in the treatment of psoriasis. The same chemokines and chemokine receptors could execute distinct functions via the regulation of both adaptive and innate immune cells in a different context. Since the discovery of ILCs, more and more emerging evidence has shown that ILCs are important for the pathogenesis of different types of inflammatory diseases. For example, NK cells are mainly responsible for CHS; ILC2s contribute more to the pathogenesis of AD; ILC3s are the major source of IL-17A, causing psoriasis (Table 1). Considering that it is still mechanistically unknown as to whether ILCs play multiple roles in allergies and chronic inflammatory diseases, the further investigation of ILCs is needed to support new novel therapies to treat inflammatory diseases. Tremendous research efforts have been undertaken on the ILCs in the lung and gut, which has expanded our knowledge of ILCs over the past 10 years. However, our current understanding of ILC biology with regard to the skin is still developing. Future investigations will need to draw a full picture of skin ILCs, including how skin ILCs are activated in different contexts and what signals they use to interact with the surrounding stromal cells (such as epithelial cells, fibroblasts, and neurons), infiltrated immune cells (T, B, DCs, macrophages, mast cells, neutrophils, eosinophils), and microorganisms. Overall, chemokines and ILCs potentially represent promising strategies for therapeutic interventions for skin inflammatory diseases.

## Figures and Tables

**Table 1:** The roles and changes of ILCs in inflamed skin.

	Cells Type	NK Cells and ILC1s	ILC2s	ILC3s
Inflammation Type				
CHS		Promote inflammation [78,80]	Negatively regulate inflammation [83]	Increased number [83]
Atopic Dermatitis		Negatively regulate inflammation [93]	Promote inflammation [95,97,114,115,116]	Increased number [117]
Psoriasis		Increased number [117]	Decreased number [113]	Promote inflammation [62,108,109,118]
